# Impact of the MHRA safety update on vitamin B_12_ testing and coding in metformin users: a retrospective primary care analysis

**DOI:** 10.1186/s12875-025-03116-1

**Published:** 2025-12-05

**Authors:** Ian Parsonage, David Wainwright, Julian Barratt

**Affiliations:** 1 Department of Health, University of Bath, Bath, UK; 2 Aston Medical School, Aston University, Birmingham, UK

**Keywords:** Vitamin B_12_, Metformin, Deficiency, Knowledge, Screening, Healthcare professional, Clinician, Awareness

## Abstract

**Background:**

Metformin is the most commonly prescribed oral treatment for type 2 diabetes mellitus (T2DM) in the UK. Long-term therapy has been linked to vitamin B_12_ deficiency, a concern recognised for decades but not consistently addressed. In June 2022, the UK Medicines and Healthcare products Regulatory Agency (MHRA) classified low vitamin B_12_ levels as a common adverse effect of metformin and advised clinicians to consider periodic testing in at-risk patients. Translating such regulatory advice into practice can be challenging, and the extent to which the guidance has influenced testing and diagnostic coding for vitamin B_12_ deficiency remains unclear. This study evaluated trends in vitamin B_12_ testing and deficiency coding in metformin-treated patients compared with the general population before and after the 2022 MHRA Drug Safety Update.

**Methods:**

A retrospective quantitative analysis was conducted using Read code data from 148,000 electronic medical records across three Primary Care Networks (PCNs) in the Southwest of England. Vitamin B_12_ testing and deficiency coding rates were compared in patients prescribed metformin and the general population across two periods: pre-guidance (2017–2021) and post-guidance (2022–2024). Welch’s t-tests were used to determine statistical significance, with *p* < 0.05 considered significant.

**Results:**

Among patients prescribed metformin, vitamin B_12_ testing rates rose from 34.5% (SD = 1.8) pre-guidance to 38.2% (SD = 0.4) post-guidance (*p* = 0.008). In the general population, testing rates increased from 12.2% to 14.7% (*p* = 0.009). However, coding for vitamin B_12_ deficiency remained unchanged at 0.25% in the metformin group and decreased from 0.072% to 0.060% in the general population, with no statistically significant difference. The post-guidance period included only two years of data, which limits the ability to assess longer-term or comparative trends between groups.

**Conclusions:**

This study demonstrated that the release of the MHRA Drug Safety Update was associated with a modest but statistically significant increase in vitamin B_12_ testing among patients prescribed metformin, paralleled by a smaller rise in testing within the general population. However, diagnostic coding practices did not change, suggesting limited translation of safety alerts into structured documentation. Further research is warranted to explore barriers and evaluate interventions to improve monitoring and coding compliance in primary care.

**Supplementary Information:**

The online version contains supplementary material available at 10.1186/s12875-025-03116-1.

## Background

Global prevalence of type 2 diabetes mellitus (T2DM) is projected to increase to 7079 individuals per 100,000 by 2030, reflecting a continued rise across all regions of the world [1]. There are concerning trends of rising prevalence in lower income countries [1]. According to the position statement from both the European Association for the Study of Diabetes (EASD) and the American Diabetes Association (ADA), metformin is the first-choice oral therapy for every patient with T2DM [2]. Even when patients are commenced on insulin, metformin therapy should be continued if tolerated [3].

Metformin belongs to a group of oral ‘anti-diabetic’ drugs called biguanides [4]. In the UK, metformin is the recommended first-line therapy for the treatment of T2DM in patients with normal renal function and is widely used [5]. Within the UK approximately 24.1 million Metformin prescriptions were dispensed [6].

In the UKPDS study [7], metformin demonstrated improved macrovascular outcomes and lower diabetes-related mortality in overweight individuals with newly diagnosed type 2 diabetes, though these findings may not be generalisable to all populations [7]. Despite these well-established benefits, long-term metformin therapy is associated with vitamin B_12_ deficiency, an effect thought to result from impaired absorption in the terminal ileum [8].

Ileal vitamin B_12_ uptake is a calcium-dependent process, and a leading hypothesis proposes that metformin interferes with this mechanism by competing with calcium at the mucosal cell membrane, thereby reducing absorption [4, 9]. Vitamin B_12_ is a water-soluble vitamin essential for DNA synthesis, normal haemopoiesis, and neurological function; deficiency therefore manifests primarily with haematological and neurocognitive features [10].

In the general adult population, the prevalence of vitamin B_12_ deficiency is estimated to be around 6% in adults younger than 60 years and approximately 20% in those older than 60 years [11], with higher rates, up to 30%, reported among people with T2DM [12]. Although these figures derive from different regions of the world, they consistently indicate an increased risk of vitamin B_12_ deficiency in those with diabetes, particularly in the context of long-term metformin use.

This association has important clinical implications. In people with T2DM, vitamin B_12_ related neuropathy can mimic diabetic peripheral neuropathy, potentially leading to misdiagnosis and delayed treatment [13]. Therefore, it is important that clinicians should remain alert to the possibility of vitamin B_12_ deficiency in patients receiving long-term metformin therapy, especially where neurological symptoms are present [13].

A relationship between long-term metformin use and vitamin B_12_ deficiency has been long discussed, and many clinicians will have come across this in clinical practice; however, until now, we have been without any official guidance on management [14]. Despite this relationship being known about since 1971, it was only in 2017 that the ADA recommended considering periodic vitamin B_12_ testing in metformin-treated patients [15]. No clear guidelines have been published regarding screening, diagnosing, and managing these deficiencies [16].

**Fig. 1:**
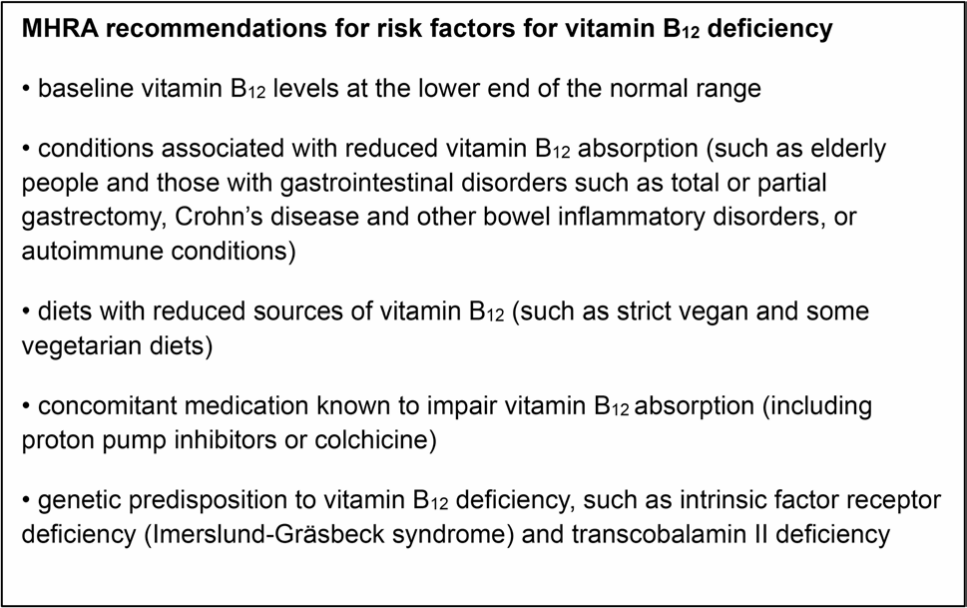
MHRA recommendations for risk factors for vitamin B_12_ deficiency [17]

In June 2022, following a European review, the MHRA issued updated advice indicating that this adverse effect is more common than earlier estimates had suggested [17]. Consequently, the prescribing information for all medicines containing metformin was revised to reflect this risk. The MHRA now classifies vitamin B_12_ deficiency as a common adverse effect of high-dose or long-term metformin, with prevalence estimates of up to 10% [17]. The updated product information includes new published advice to healthcare professionals to test vitamin B_12_ levels in those presenting with anaemia or neuropathy and that periodic vitamin B_12_ monitoring should be considered in patients with risk factors (see Fig. 1) for vitamin B_12_ deficiency [17].

Evidence linking metformin to vitamin B_12_ deficiency has long been circulated in specialist diabetes and academic literature. An MHRA safety communication was issued to raise clinician awareness of this adverse effect. However, the extent to which such regulatory guidance translates into measurable change in primary care remains uncertain [18]. Therefore, the aim of this study is to explore the overall incidence of coding vitamin B_12_ deficiency in primary care settings and if there was any impact on vitamin B_12_ monitoring in metformin-treated patients in a primary care setting, following the change in MHRA guidance [17].

## Method

### Study design

This quantitative study employed a retrospective design, analysing Read-coded data relating to vitamin B₁₂ blood tests and coded diagnoses of vitamin B_12_ deficiency. The data were extracted from the electronic medical record systems used by general practices within the authors’ local Primary Care Networks (PCNs). As all eligible patient records meeting the inclusion criteria were included in the analysis, no additional sampling strategy was necessary.

### Study setting

A PCN is a group of General Practice (GP) practices that work together with other local health and care services to provide more coordinated care for their communities. A GP surgery (practice) in the UK refers to a primary care medical practice where general practitioners (family doctors) and associated healthcare professionals provide first-contact, continuous, and comprehensive care to registered patients.

The study utilised a geographical area containing three PCNs. It was chosen as it provided a sample size that was realistic for the resources for the study and utilised the same computer system for the electronic medical record meaning only one audit algorithm needed to be designed (helping with the reliability of the data).

### Study participants and sampling

The study data was collected from nine of the GP surgeries that were part of the 3 PCNs. The 3 PCNs represented a patient population size of approx.148,000 which included a mixture of different socio-economic backgrounds and both a rural and urban population (See Table 1 for details).

**Table 1:** Local statistics compared to national statistics from the Office of National Statistics [19]

Indicator	Torbay (Unitary Authority)	England (Average)
Median Age	43 years	40 years
Ethnicity (% White)	~96.1 % (White)	81.0 % (White, all categories)
Employment Rate (working age)	76.6 % (ONS 2021)	~74.9 %
Unemployment Rate (ages 16 +)	2.1 % (2021 county data)	2.8 %
Median Salary (full-time employees)	Approximately £28,000	≈ £33,000
Deprivation (IMD 2019 rank)	48th most deprived local authority of 317 in England	—
Rural/Urban Mix	Predominantly urban coastal area encompassing Paignton and Brixham, with some semi-rural communities	—

As all data required via the inclusion criteria (Table 2) will be aggregated from the electronic medical record, no sampling technique will be required.

**Table 2:** Inclusion/exclusion criteria for searches

Criteria Type	Description
Inclusion	- Patients aged 18 years or older at the time of data extraction.
	- Registered with a GP practice within one of the three participating PCNs in Southwest England.
	- Had active electronic health records available for the study periods (2017–2021 and/or 2022–2024).
	- For the metformin group only:
	- Have a coded diagnosis of T2DM
	- Received at least two consecutive (no gap in prescribing allowed) prescriptions for metformin.
	- On metformin therapy for at least 12 months (with consecutive prescriptions for those previous 12 months) before the end of each analysis period.
Exclusion	- Incomplete or missing electronic medical records for the relevant study periods.
	- Not registered in participating PCNs.
	- Under the age of 18yrs old at the time of extraction.

### Participants’ recruitment

There was no transfer of any personal information, which means patient’s notes were not accessed individually, the aggregated data was extracted anonymously from medical records using Read code data.

Consent was gained to collect the data from the Clinical Director (CD) of each participating organisation. As no personal identifiable information was collected and no electronic medical notes were individually accessed, individual consent of patients was not deemed necessary. This was agreed both by the University of Bath Ethics board and the Health Regulatory Authority. Once agreed the PCN CD discussed it with each surgery individually and written consent was gained from each surgery as they are legally the data controller for their medical records.

### Data collection

The study analysed Read code data from the electronic medical notes and specifically looked at vitamin B_12_ level testing in adult patients who had more than 1 year of metformin use, 5 years prior to the guidance publication (June 2022) and who had a record of at least two consecutive prescriptions of metformin, to establish compliance. The same data was collected for the general population (excluding the patients on metformin) to act as a comparison group. The same data was collected, but for the time period after June 2022 (when the MHRA alert was released) with again data being collected for patients on metformin and the general population.

The data collection tool was also run for the Read codes related to low vitamin B_12_ serum levels and vitamin B_12_ deficiency for patients on metformin and the general population across both time periods, to explore if this is coded in the medical notes (as this puts the patients in the high-risk category as per the MHRA guidelines).

The primary outcome was to explore if the incidence of vitamin B_12_ testing increased in the patient population on metformin before and after the guidance was issued. The secondary outcome was to explore if the incidence of vitamin B_12_ deficiency coding in patients on metformin changed after the MHRA guidance was released.

### Data analysis

Summary statistics were initially used to describe the data collected. To explore the differences in the percentage of patients coded for vitamin B_12_ deficiency and the percentage of patients who had received a blood test for vitamin B_12_ before and after 2022, an independent samples Welch’s t-tests was performed for each group. Welch’s t-test was chosen due to the small sample sizes and potential violation of equal variance assumptions between the time periods. A two-tailed significance level of *p* < 0.05 was considered indicative of statistical significance.

Formal testing for skewness was not undertaken due to the small number of annual data points available, particularly in the post-guidance period (*n* = 2), which limits meaningful interpretation of distribution shape. Given the nature of the aggregated percentage data and the use of Welch’s t-test, a method robust to non-normality and unequal variances, this was considered acceptable within the context of the analysis.

## Results

### Blood tests

Analysis of Read code data across three PCNs revealed an increase in vitamin B_12_ testing among patients prescribed metformin following the publication of the MHRA Drug Safety Update [17]. During the 2017–2021 period, an average of 4,229 patients on metformin were recorded annually, with approximately 1,453 individuals (34.5%) receiving a vitamin B_12_ blood test each year. In the period 2022–2024, although the average number of metformin users remained similar at 4,188 per year, the number of patients undergoing vitamin B_12_ testing increased to around 1,599 annually (38.2%). The proportion of patients who received a vitamin B_12_ blood test increased from 34.5% (SD 1.8) to 38.2% (SD 0.4). Welch’s t-test demonstrated a statistically significant difference between the two time periods (t(4.66) = − 4.38, *p* = 0.008).

**Fig. 2:**
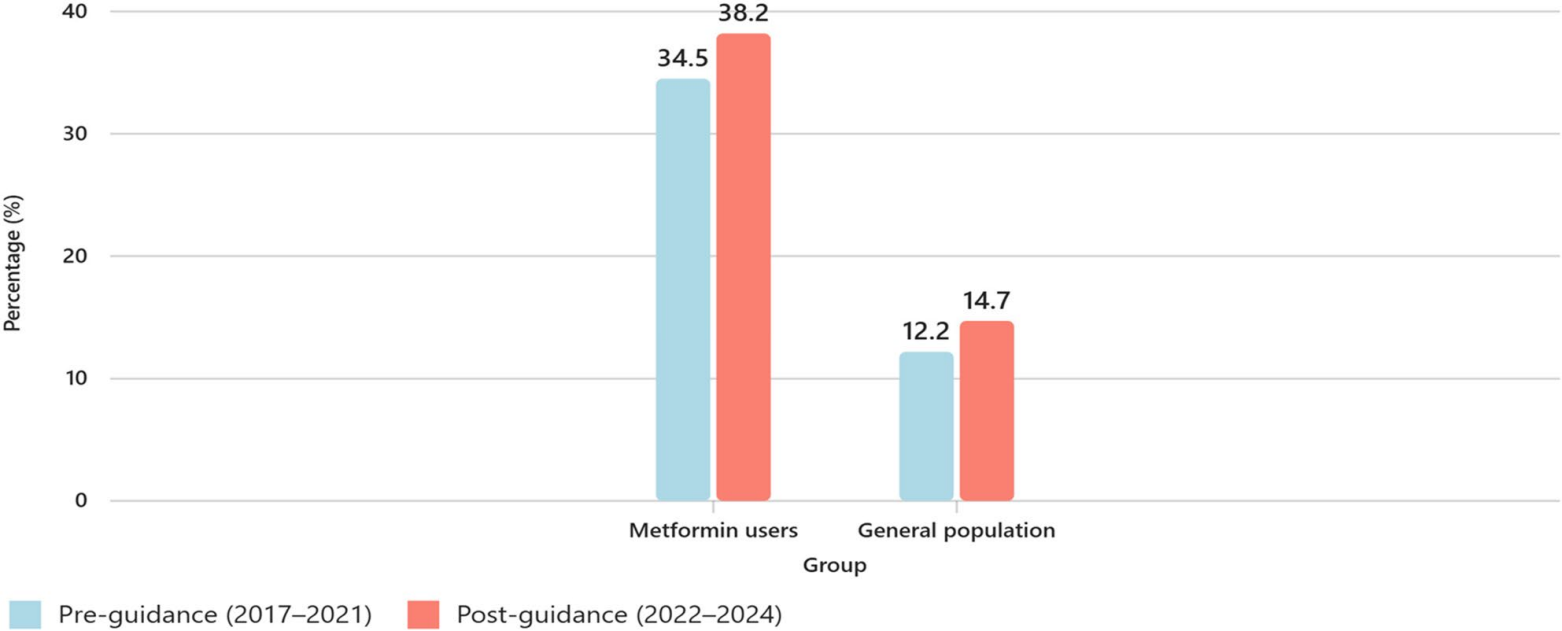
Graph demonstrating % difference in testing for vitamin B_12_ deficiency pre and post MHRA guidance in both populations

A similar trend was observed in the general population not taking metformin, where the average number of individuals tested each year rose from 4,733 (12.2% of the eligible population) before the MHRA Drug Safety Update [17] to 5,326 (14.7%) after its release. In the general population (excluding metformin users), the mean annual proportion of patients tested for vitamin B_12_ rose from 12.2% (SD 1.3) to 14.7% (SD 0.4). This difference was also statistically significant (t(5.05) = − 4.10, *p* = 0.009). The mean annual patient numbers for this group were 38,800 during 2017–2021 and 38,750 during 2022–2024. This is summarised below in Table 3; Fig. 2.

**Table 3:** Vitamin B12 testing rates before and after MHRA guidance

Group	*n* (mean annual)	Pre-guidance 2017–2021 % (SD)	Post-guidance 2022–2024 % (SD)	Mean Difference (%)	Welch t (df)	*p* value
Metformin users	4,229	34.5 (1.8)	38.2 (0.4)	+3.7	–4.38 (4.66)	0.008
General population	38,800	12.2 (1.3)	14.7 (0.4)	+2.5	–4.10 (5.05)	0.009

### Coding

The Read code entries revealed that the rate of coding for vitamin B_12_ deficiency among patients prescribed metformin remained low and unchanged following the MHRA Drug Safety Update [17]. In the years 2017–2021, an average of 4,229 patients were prescribed metformin annually, with approximately 11 patients per year (0.250%) receiving a formal Read code indicating vitamin B_12_ deficiency. This figure remained consistent in the 2022–2024 period, with a comparable average of 4,188 patients on metformin and again just 11 patients annually (0.250%) being coded for deficiency. Read-coded entries for vitamin B_12_ deficiency were examined for both cohorts. Among patients prescribed metformin, the mean annual proportion coded for deficiency was 0.25% (SD 0.05) in 2017–2021 and 0.25% (SD 0.08) in 2022–2024. Welch’s t-test showed no statistically significant difference between the two periods (t(2.75) = − 0.004, *p* = 0.997).

**Fig. 3:**
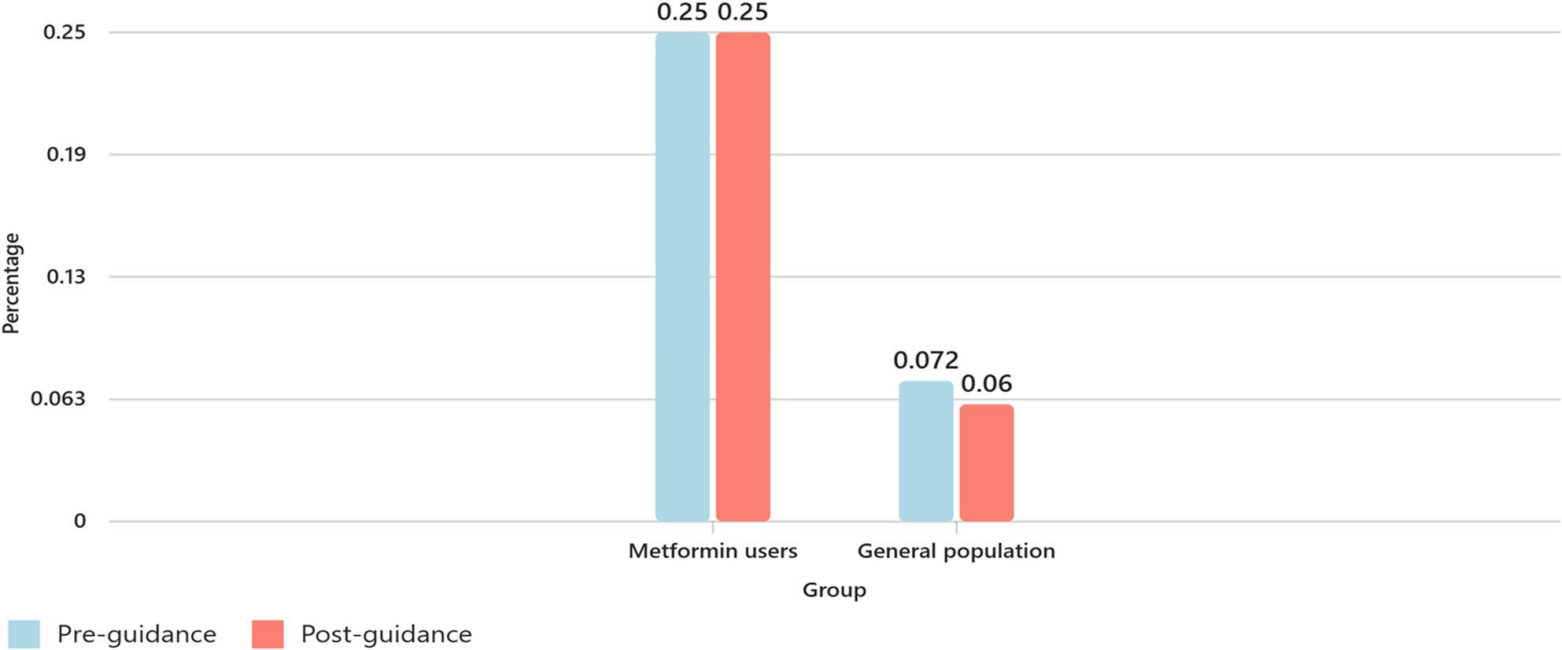
Graph demonstrating % difference in coding for vitamin B_12_ deficiency pre and post MHRA guidance in both populations

In the general population (excluding metformin users), coding rates were even lower: on average, 28 patients per year (0.072%) were coded for deficiency before the MHRA guidance [17], compared with 26 patients annually (0.060%) after the update. In the general population, coding for vitamin B_12_ deficiency was 0.072% (SD 0.024) during 2017–2021 and 0.060% (SD 0.005) during 2022–2024. This difference was not statistically significant (t(4.56) = 1.05, *p* = 0.348). This is summarised below in Table 4; Fig. 3.

**Table 4:** Vitamin B12 deficiency coding before and after MHRA guidance

Group	*n* (mean annual)	Pre-guidance % (SD)	Post-guidance % (SD)	Mean Difference (%)	Welch t (df)	*p* value
Metformin users	4,229	0.25 (0.05)	0.25 (0.08)	0.00	–0.004 (2.75)	0.997
General population	38,800	0.072 (0.024)	0.060 (0.005)	–0.012	1.05 (4.56)	0.348

## Discussion

The results of this study demonstrated above suggest a statistically significant increase in vitamin B_12_ blood testing in patients on metformin following the MHRA safety alert in 2022 [17] in the geographical area involved in this study. Whilst the proportion of tested patients rose from 34.5% to 38.2%, this improvement, though statistically valid (*p* = 0.008), represents only a modest shift in clinical behaviour. Despite the observed improvement in testing rates, the overall proportion of metformin users tested remains below 40%, indicating that routine B_12_ monitoring is not yet embedded in standard clinical practice. This is consistent with findings from other similar studies [20, 21] which found that only 31% of patients on metformin, over four years had ever received a vitamin B_12_ blood test.

Additionally, the concurrent increase in testing within the general population (from 12.2% to 14.7%, *p* = 0.009) suggests that the rise may reflect a broader trend towards more routine blood test ordering in primary care, rather than a targeted response to the MHRA Drug Safety Update [17]. This is consistent with UK-wide data showing rising diagnostic testing volumes across all major analytes over the last decade, with what appears to be vitamin B_12_ as being no exception [22, 23].

Even though, both the metformin and general population groups demonstrated statistically significant increases in vitamin B_12_ testing following the MHRA alert (*p* = 0.008 and *p* = 0.009, respectively), the present dataset was insufficient to determine whether the magnitude of change between the two groups differed significantly. Numerically, the increase was slightly greater among metformin users (3.7%) than the general population (2.5%), but this difference could not be confirmed statistically due to the limited number of post-guidance data points.

Nonetheless, this study has only found a modest statistically significant increase in B_12_ testing after the MHRA Drug Safety Update [17], which supports the impression that regulatory safety updates can exert some influence on practice, albeit modest. This is further supported in literature in which prior studies have reported similar effects following national guidance changes [24, 25]. Though, research has shown that successful translation of guidance into routine practice is often dependent on accompanying education, audit, or system-level prompts [26]. In this study, there is no indication that practices had access to clinical decision support or embedded alerts encouraging B_12_ testing in high-risk patients.

Interestingly, the increase in testing was not mirrored by a corresponding rise in diagnosis of vitamin B_12_ deficiency. The findings suggest that either vitamin B_12_ deficiency remains under-identified in this group, or that testing is being undertaken without clear action on the results. This is not unique to B_12_ deficiency. Other studies have shown similar under-coding in primary care, often linked to lack of time, diagnostic uncertainty, or reliance on free-text entries rather than structured Read codes [27].

The findings of this study are consistent with other respective studies. Other authors [21, 28] noted a low coding rate, though this study reports a considerably lower rate than the other studies. The coding rates reported in the other studies were between 0.5% and 2.6%. Additionally, one of these studies reported a coding rate of 2.6% whilst the actual number of patients with vitamin B_12_ deficiency found in the study was 8.4% [21].

Based on the data above, there is a risk that subtle, or early-stage deficiency is not being recognised at all. The low rate of vitamin B_12_ deficiency coding raises concerns about missed or subclinical cases. Comparable studies suggest that early B_12_ deficiency, particularly in patients with borderline results, is frequently overlooked unless symptoms are overt [28]. This is especially problematic in patients with T2DM, where B_12_ related neuropathy may be mistakenly attributed to diabetic neuropathy, delaying appropriate intervention [13].

The study’s findings reflect known challenges in translating policy into practice. The PARIHS framework (Promoting Action on Research Implementation in Health Services) emphasises the interplay between evidence, context, and facilitation in successful implementation [29]. In this case, the strength of the evidence linking metformin with B_12_ deficiency is well established, but its implementation appears constrained by potential contextual factors such as competing clinical priorities, lack of system prompts, and possible limited training. It has been argued that research consistently shows that passive dissemination of guidelines, such as publication of alerts, has minimal effect unless accompanied by active implementation strategies [26, 30].

### Strengths and limitations

This study has several methodological strengths. It draws on routinely collected primary care data from multiple practices within three PCNs, providing a real-world picture of testing and coding behaviour across a diverse patient population The inclusion of all eligible patients meeting the study criteria avoided sampling bias and ensured a comprehensive assessment of population-level testing behaviour. The use of Read-coded data allowed consistent identification of both vitamin B_12_ testing and diagnostic coding over time, supporting reproducibility and comparability with other datasets. The inclusion of a general population comparator group provided an additional internal control, strengthening the interpretation of observed trends among metformin users.

Nonetheless, the findings should be interpreted considering several limitations. The data on metformin dose, treatment duration, and other individual risk factors were not available in a consistent coded format across practices and were therefore not included in this analysis. The post MHRA Drug Safety Update [17] time frame includes only two full years (2022–2024), limiting the power to detect longer-term behavioural trends or seasonal variations. Additionally, there is not enough data points post guidance to accurately analyse if the joint upward trend in blood tests undertaken, was statistically significant between the metformin and general population group. Furthermore, while the inclusion of a comparison group (the general population) strengthens the internal validity, the study cannot definitively attribute causality to the MHRA Drug Safety Update [17] without accounting for other contemporaneous factors, such as changes in local policies.

## Conclusion

This study demonstrates that the MHRA Drug Safety Update [17] was associated with a modest but statistically significant increase in vitamin B_12_ testing among patients prescribed metformin within primary care. Although this suggests that regulatory alerts can positively influence clinical behaviour, the absolute improvement was small, and fewer than 40% of patients on metformin underwent vitamin B_12_ testing. The simultaneous rise in testing among the general population further indicates that the observed increase may partly reflect wider trends in diagnostic activity rather than a targeted response to the MHRA guidance.

Importantly, diagnostic coding of vitamin B_12_ deficiency did not increase following the guidance, suggesting a continued gap between testing and formal documentation. This disconnect highlights potential challenges in translating safety updates into consistent coding and management practices, with implications for both patient safety and data quality.

Taken together, these findings illustrate that while safety communications can prompt incremental change, they alone are insufficient to drive systematic improvement. Building on recent evidence that implementation of MHRA guidance across UK primary care remains variable [18], this study reinforces the need for multifaceted strategies, such as electronic alerts, audit and education, to embed new safety recommendations into daily clinical practice.

### Implications for practice and policy

These findings highlight that routine vitamin B_12_ monitoring in patients prescribed metformin remains inconsistent within primary care, despite national safety guidance. Future research planned by the authors will explore the level of clinician awareness regarding the MHRA Drug Safety Update [17] and explore the contextual barriers and facilitators that influence implementation of vitamin B_12_ monitoring in everyday practice. Understanding these behavioural and organisational factors will be essential to designing effective strategies to close the evidence practice gap. Further research could explore if incorporating electronic prompts within prescribing systems and annual diabetes review templates support more consistent testing.

## Supplementary Information

Supplementary Material 1.

## Data Availability

Data is provided within the manuscript or supplementary information files.

## Abbreviations

ADA: American Diabetes Association
B12: Vitamin B12
CD: Clinical Director
DNA: Deoxyribonucleic acid
EASD: European Association for the Study of Diabetes
EMR: Electronic medical record
GP: General practitioner
HRA: Health Research Authority
IRAS: Integrated research application system
MHRA: Medicines and Healthcare products Regulatory Agency
NICE: National Institute for Health and Care Excellence
NIHR: National Institute for Health and Care Research
ONS: Office for National Statistics
PARIHS: Promoting Action on Research Implementation in Health Services
PCN: Primary Care Network
SD: Standard deviation
T2DM: Type 2 Diabetes Mellitus
UKPDS: United Kingdom Prospective Diabetes Study

## References

[1] Khan MAB, Hashim MJ, King JK, Govender RD, Mustafa H, Al Kaabi J. Epidemiology of type 2 diabetes – global burden of disease and forecasted trends. J Epidemiol Glob Health. 2019;10(1):107. 10.2991/jegh.k.191028.001. 10.2991/jegh.k.191028.001 PMC7310804 32175717

[2] Davies MJ, Aroda VR, Collins BS, Gabbay RA, Green J, Maruthur NM, et al. Management of hyperglycemia in type 2 diabetes, 2022. A consensus report by the American Diabetes Association (ADA) and the European Association for the Study of Diabetes (EASD). Diabetes Care. 2022;45(11):2753–86. 10.2337/dci22-0034. 36148880 10.2337/dci22-0034 PMC10008140

[3] Inzucchi SE, Bergenstal RM, Buse JB, Diamant M, Ferrannini E, Nauck M, et al. Management of hyperglycemia in type 2 diabetes: a patient-centered approach. Diabetes Care. 2012;35(6):1364–79. 10.2337/dc12-0413. 22517736 10.2337/dc12-0413 PMC3357214

[4] Al Zoubi MS, Al Kreasha R, Aqel S, Saeed A, Al-Qudimat AR, Al-Zoubi RM. Vitamin B12 deficiency in diabetic patients treated with metformin: a narrative review. Irish Journal of Medical Science (1971 -). 2024;193(4):1827–35. 10.1007/s11845-024-03634-4. 10.1007/s11845-024-03634-4 PMC11294377 38381379

[5] National Institute for Health and Care Excellence (NICE). Type 2 diabetes in adults: management (NG28). 2022. Available at: https://www.nice.org.uk/guidance/ng28 [Accessed 25 June 2025]. 32023018

[6] Statista. Number of metformin prescriptions in England 2010–2023. 2024. Available at: https://www.statista.com/statistics/number-of-prescriptions-metformin-uk/ [Accessed 25 June 2025].

[7] UK Prospective Diabetes Study Group. Effect of intensive blood-glucose control with Metformin on complications in overweight patients with type 2 diabetes. Lancet. 1998;352(9131):854–65. 9742977

[8] Biemans E, Hart HE, Rutten GEHM, Cuellar Renteria VG, Kooijman-Buiting AMJ, Beulens JWJ. Cobalamin status and its relation with depression, cognition and neuropathy in patients with type 2 diabetes mellitus using metformin. Acta Diabetol. 2014;52(2):383–93. 10.1007/s00592-014-0661-4. 25315630 10.1007/s00592-014-0661-4

[9] Bauman WA, Shaw S, Jayatilleke E, Spungen AM, Herbert V. Increased intake of calcium reverses vitamin B12 malabsorption induced by metformin. Diabetes Care. 2000;23(9):1227–31. 10.2337/diacare.23.9.1227. 10977010 10.2337/diacare.23.9.1227

[10] Kibirige D, Mugenyi L, Kaddu M, Opio C, Lalitha R, Mutebi E, et al. Vitamin B12 deficiency among adult diabetic patients in Uganda: relation to glycaemic control and haemoglobin concentration. Journal of Diabetes & Metabolic Disorders. 2015. 10.1186/s40200-016-0250-x. 10.1186/s40200-016-0250-x PMC4962419 27468410

[11] Hunt A, Harrington D, Robinson S. Vitamin B12 deficiency. BMJ. 2014;349(sep04 1):g5226–g5226. 10.1136/bmj.g5226. 25189324 10.1136/bmj.g5226

[12] Nervo M, Lubini A, Raimundo FV, Faulhaber GA, Leite C, Fischer LM, et al. Vitamin B12 in metformin-treated diabetic patients: a cross-sectional study in Brazil. Rev Assoc Med Bras (1992). 2011;57(1):46–9. 21390459

[13] Alvarez M, Sierra OR, Saavedra G, Moreno S. Vitamin B12 deficiency and diabetic neuropathy in patients taking metformin: a cross-sectional study. Endocr Connect. 2019;8(10):1324–9. 10.1530/ec-19-0382. 31518991 10.1530/EC-19-0382 PMC6790897

[14] Davies J. Metformin and vitamin B12: long overdue guidance. J Diabetes Nurs. 2022;26(5):1–4.

[15] Carracher AM, Marathe PH, Close KL. International Diabetes Federation 2017. J Diabetes. 2018;10(5):353–6. 10.1111/1753-0407.12644. 29345068 10.1111/1753-0407.12644

[16] Infante M, Leoni M, Caprio M, Fabbri A. Long-term metformin therapy and vitamin B12 deficiency: an association to bear in mind. World J Diabetes. 2021;12(7):916–31. 10.4239/wjd.v12.i7.916. 34326945 10.4239/wjd.v12.i7.916 PMC8311483

[17] Medicines and Healthcare products Regulatory Agency (MHRA). Metformin and vitamin B12 deficiency: Drug safety update. 2022. Available at: https://www.gov.uk/drug-safety-update/metformin-risk-of-vitamin-b12-deficiency [Accessed 25 June 2025].

[18] Parsonage I, Wainwright D, Barratt J. Vitamin B12 deficiency in long-term metformin use and clinician awareness: a scoping review protocol. BMJ Open. 2025;15(7):e101016. 10.1136/bmjopen-2025-101016. 40701582 10.1136/bmjopen-2025-101016 PMC12306209

[19] Office for National Statistics. Census. London: Office for National Statistics. Available from: https://www.ons.gov.uk/census. Cited 19 Oct 2025.

[20] Treadwell JS, Wong G, Milburn-Curtis C, Feakins B, Greenhalgh T. GPs’ understanding of the benefits and harms of treatments for long-term conditions: an online survey. BJGP Open. 2020;4(1):bjgpopen20X101016. 10.3399/bjgpopen20x101016. 32127362 10.3399/bjgpopen20X101016 PMC7330197

[21] Qureshi S, Ainsworth A, Winocour P. Metformin therapy and assessment for vitamin B12 deficiency: is it necessary? Pract Diab. 2011;28(7):302–4. 10.1002/pdi.1619.

[22] Jones T, Patel R, Elwenspoek MMC, Watson JC, Mann E, Alsop K, et al. Variation in laboratory testing for patients with long-term conditions: a longitudinal cohort study in UK primary care. BJGP Open. 2022;7(1):BJGPO.2022.0139. 10.3399/bjgpo.2022.0139. 10.3399/BJGPO.2022.0139 PMC10354328 36693759

[23] O’Sullivan JW, Stevens S, Hobbs FDR, Salisbury C, Little P, Goldacre B, Bankhead C, Aronson JK, Perera R, Heneghan C. Temporal trends in use of tests in UK primary care, 2000-15: retrospective analysis of 250 million tests. 2018. BMJ. p.k4666. Available from: 10.1136/bmj.k4666. 10.1136/bmj.k4666 PMC6260131 30487169

[24] Dusetzina SB, Higashi AS, Dorsey ER, Conti R, Huskamp HA, Zhu S, Garfield CF, Alexander GC. Impact of FDA drug risk communications on health care utilization and health behaviors: a systematic review. Med Care. 2012;50(6):466–78. 10.1097/MLR.0b013e318245a160. 22266704 10.1097/MLR.0b013e318245a160 PMC3342472

[25] Morrow RL, Mintzes B, Souverein PC, De Bruin ML, Roughead EE, Lexchin J, et al. Influence of drug safety advisories on drug utilisation: an international interrupted time series and meta-analysis. BMJ Qual Saf. 2022;31(3):179–90. 10.1136/bmjqs-2021-013910. 35058332 10.1136/bmjqs-2021-013910 PMC8899478

[26] Greenhalgh T, Robert G, Macfarlane F, Bate P, Kyriakidou O. Diffusion of innovations in service organizations: systematic review and recommendations. Milbank Q. 2004;82(4):581–629. 10.1111/j.0887-378X.2004.00325.x. 15595944 10.1111/j.0887-378X.2004.00325.x PMC2690184

[27] Tulloch JS, Beadsworth MB, Vivancos R, Radford AD, Warner JC, Christley RM. GP coding behaviour for non-specific clinical presentations: a pilot study. BJGP Open. 2020;4(3):bjgpopen20X101050. 10.3399/bjgpopen20x101050. 32636202 10.3399/bjgpopen20X101050 PMC7465576

[28] Alshammari A, Iqbal R, Baksh I. Vitamin B12 deficiency and the knowledge and practice of physicians regarding screening for vitamin B12 deficiency among type 2 diabetic patients on metformin in selected hospitals in Riyadh, Saudi Arabia. J Fam Med Prim Care. 2019;8(7):2306. 10.4103/jfmpc.jfmpc_416_19. 10.4103/jfmpc.jfmpc_416_19 PMC6691434 31463247

[29] Kitson AL, Rycroft-Malone J, Harvey G, McCormack B, Seers K, Titchen A. Evaluating the successful implementation of evidence into practice using the PARiHS framework: theoretical and practical challenges. Implement Sci. 2008. 10.1186/1748-5908-3-1. 18179688 10.1186/1748-5908-3-1 PMC2235887

[30] Grol R, Grimshaw J. From best evidence to best practice: effective implementation of change in patients’ care. Lancet. 2003;362(9391):1225–30. 10.1016/s0140-6736(03)14546-1. 14568747 10.1016/S0140-6736(03)14546-1

